# High-Performance Image Acquisition and Processing for Stereoscopic Diagnostic Systems with the Application of Graphical Processing Units

**DOI:** 10.3390/s22020471

**Published:** 2022-01-08

**Authors:** Piotr Perek, Aleksander Mielczarek, Dariusz Makowski

**Affiliations:** Department of Microelectronics and Computer Science (DMCS), Lodz University of Technology (TUL), 93-005 Lodz, Poland; aleksander.mielczarek@p.lodz.pl (A.M.); dariusz.makowski@p.lodz.pl (D.M.)

**Keywords:** stereoscopy, 3D, disparity map, uncalibrated rectification, graphics processing units, GPU, diagnostic system

## Abstract

In recent years, cinematography and other digital content creators have been eagerly turning to Three-Dimensional (3D) imaging technology. The creators of movies, games, and augmented reality applications are aware of this technology’s advantages, possibilities, and new means of expression. The development of electronic and IT technologies enables the achievement of a better and better quality of the recorded 3D image and many possibilities for its correction and modification in post-production. However, preparing a correct 3D image that does not cause perception problems for the viewer is still a complex and demanding task. Therefore, planning and then ensuring the correct parameters and quality of the recorded 3D video is essential. Despite better post-production techniques, fixing errors in a captured image can be difficult, time consuming, and sometimes impossible. The detection of errors typical for stereo vision related to the depth of the image (e.g., depth budget violation, stereoscopic window violation) during the recording allows for their correction already on the film set, e.g., by different scene layouts and/or different camera configurations. The paper presents a prototype of an independent, non-invasive diagnostic system that supports the film crew in the process of calibrating stereoscopic cameras, as well as analysing the 3D depth while working on a film set. The system acquires full HD video streams from professional cameras using Serial Digital Interface (SDI), synchronises them, and estimates and analyses the disparity map. Objective depth analysis using computer tools while recording scenes allows stereographers to immediately spot errors in the 3D image, primarily related to the violation of the viewing comfort zone. The paper also describes an efficient method of analysing a 3D video using Graphics Processing Unit (GPU). The main steps of the proposed solution are uncalibrated rectification and disparity map estimation. The algorithms selected and implemented for the needs of this system do not require knowledge of intrinsic and extrinsic camera parameters. Thus, they can be used in non-cooperative environments, such as a film set, where the camera configuration often changes. Both of them are implemented with the use of a GPU to improve the data processing efficiency. The paper presents the evaluation results of the algorithms’ accuracy, as well as the comparison of the performance of two implementations—with and without the GPU acceleration. The application of the described GPU-based method makes the system efficient and easy to use. The system can process a video stream with full HD resolution at a speed of several frames per second.

## 1. Introduction

Stereopsis—the natural ability of humans and animals to see the 3D world [1,2]—still presents a challenge to emulate in the electronic and IT world. Stereoscopy, a technique imitating three-dimensional vision, allows reproducing not only the shape and colour of objects, but also their mutual position in space and distance from the observer. Thanks to this, 3D technology enables new means of expression and allows providing the audience with impressions unreachable with Two-Dimensional (2D) imaging technology. For this reason, cinematography has been interested in 3D imaging almost from its introduction. The current rapid development of electronics, and hence video recording and processing technologies, gave another opportunity for the development of 3D technology and significantly expanded the areas of its application. It can be useful in education, science, engineering, and medicine, but this paper focuses on broadly understood entertainment applications (cinematography, games, virtual reality, etc.).

The application of current technologies such as high-resolution cameras and high-performance software tools for video analysis and post-processing causes stereoscopic films to be more realistic and impressive. The 3D displaying techniques previously available only in cinemas (e.g., polarised 3D system in IMAX) are now implemented in standard TV sets, so 3D content is readily and commonly available. However, the production of 3D video is a complex process, more challenging than in the case of 2D. People responsible for creating and supervising the 3D video recording and post-production need to know the new issues and restrictions coming together with the transition from 2D to 3D content.

The most important new aspect related to 3D video quality assessment is visual comfort. An incorrect 3D image may not only be aesthetically poor, but may also harm the viewer’s well-being. The key sources of visual fatigue are vertical disparity, vergence-accommodation conflicts, stereoscopic window violation, or exceeding the horizontal disparity limits [3]. Therefore, various automated methods of assessing the quality of a 3D image have been proposed [4]. Many of them are based on the determination and analysis of horizontal and vertical disparities [4,5,6]. The horizontal disparity is significant, as it is a good measure informing about the position of an object in space. It is assumed that objects in a correct 3D image should be within a specific disparity range. The range, known as the comfort viewing zone, varies depending on the camera used for video recording and the planned display system. For this reason, the depth budget should be planned at the stage of creating the film [7], and then, the assumed limits should be monitored throughout the production cycle, starting with recording.

The image registration process is the crucial stage in creating 3D content that affects the quality of the final material. The 3D quality assurance and control should start from the very beginning of the movie production—from the raw image recording. Even though the raw data go through post-production, where some errors can be fixed or minimised, it is essential to ensure that it is high quality and error free. Its further processing and improvement can be costly and time consuming, and sometimes, it can be even impossible to fix some errors in the raw input image.

Electronic and IT tools for monitoring the quality, especially the disparity limits, can significantly facilitate the work of a film crew and enable quick and reliable detection of errors during 3D video recording. Exemplary systems supporting the recording crew were proposed in [7,8,9]. The Stereoscopic Analyzer (STAN) is an image-based assistance tool dedicated to 3D video shooting [8,9]. It supports the mechanical alignment of the stereoscopic cameras, allows correcting the distortions in the recorded image, and enables determining the current depth window. The depth budget analysis is performed based on selected feature points. A similar functionality is offered by the real-time stereoscopic analyser proposed in [7]. The analyser monitors the horizontal disparity distribution and generates warnings if the budget is exceeded.

The system presented in this paper is dedicated to supporting the film crew, especially stereographers, in 3D video quality control and assessment (see Section 2). It can be easily integrated with 3D cameras and used directly on the film set during the recording process. The system’s main goal is to calculate and visualise a complete disparity map for the recorded scene. The map is appropriately coloured to inform about the screen plane and the assumed depth limits. The system implements efficient GPU-based image-processing algorithms to estimate and analyse the disparity map of the 3D video (see Section 3). The results of evaluating the algorithms in terms of accuracy and computational performance are shown in Section 5.

### 1.1. Use Cases

The research presented in this paper aimed to propose a complete image analysis method for live monitoring of the 3D video quality during professional recordings on a film set. The proposed algorithms, prototype diagnostic system, and software implementation were developed to ensure two main functionalities important during 3D video recording:

- Support during calibration of the stereoscopic rig to precisely adjust the optical paths of two cameras and eliminate undesirable vertical disparity as one of the main sources of visual fatigue;
- Disparity map estimation and 3D depth analysis to avoid depth budget/range exceeding and stereoscopic window violation.

Professional systems for recording 3D movies consist of two independent film cameras mounted on a dedicated mechanical structure called a stereoscopic rig. The rig allows changing the relative position of the cameras and, most importantly, setting the selected convergence. Due to their mechanical structure, rigs are divided in parallel (where the cameras are installed next to each other) and perpendicularly (where the cameras are mounted perpendicularly using a semi-transparent mirror). Camera calibration aims to align the optical paths of both cameras to obtain a consistent and correct stereoscopic image. This procedure must always be performed at the beginning of the shooting day and repeated many times during the shooting. For this reason, it should not be a long process and at the same time precise, because the quality of the recorded image depends to a large extent on it. The proposed calibration method is based on the dedicated reference board with markers detectable by the algorithms implemented in the system (details can be found in [10]).

The paper focuses on the second functionality: disparity map estimation for the needs of 3D depth monitoring on the movie set while recording the stereoscopic image. The analysis at the first stage of 3D video production aims to avoid visual fatigue sources such as stereoscopic window violation and vergence-accommodation conflicts. In general, the estimation and analysis of the disparity map can be performed in post-production. However, errors detected at this stage cannot be fixed in many cases or their repairing may entail high time or monetary costs. Therefore, the disparity map should be estimated and monitored on the movie set during stereoscopic video recording.

### 1.2. Requirements

The nature of the work on the film set (high dynamics of the changes, time pressure, unfavourable conditions) imposes specific requirements and expectations related to the auxiliary systems used during recording. They should improve and support the activities performed on the film set and not complicate them even further. Only in such a case their application is justified and brings noticeable benefits to the film crew.

Therefore, the diagnostic system supporting disparity map estimation and analysis should be easy to use, which means requiring as little configuration activities and user interaction as possible. The system also should not require additional connections or signals to be provided mainly for its purposes. Ideally, it should produce valuable results only from signals that are already standardly available on a film set. As a diagnostic tool for the film crew, the system should generate a disparity map that faithfully reflects the cameras’ image without distortions and losses. The results, indicators, and meters produced by the system should be easy to unambiguously interpret. The system should offer capacity sufficient to process video streams typical of modern cinema (at least full HD resolution) on an ongoing basis (at least one frame per second).

## 2. Image Acquisition and Processing System Architecture

**Figure 1:**
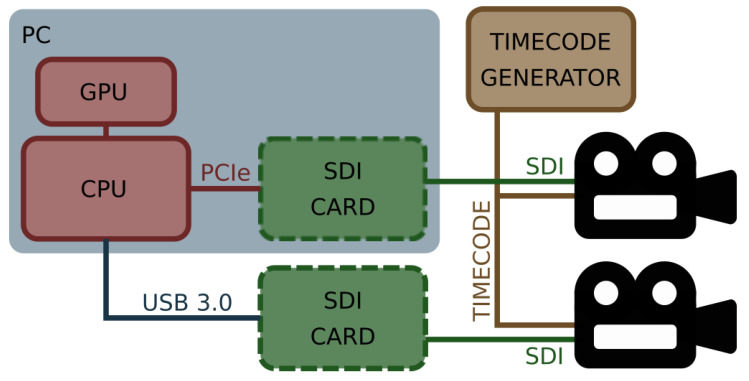
Architecture of the GPU-based diagnostic system for stereoscopic rigs.

Based on the above requirements and discussions with the creators of 3D movies, two different system architectures were considered. One of them is based on the computer equipped with a Central Processing Unit (CPU), GPU, and extension cards for image acquisition. Another one assumes the development of a dedicated electronic device based on an Field-Programmable Gate Array (FPGA) circuit. Considering that the development of firmware for FPGA devices is complicated and time consuming, it was decided that the architecture of the system be based on the CPU and GPU. This solution is more flexible, easier to use, maintain, and extend, and gives more freedom in system development. The architecture of the developed system is presented in Figure 1.

As already mentioned, the heart of the system is a computer equipped with a CPU and GPU accelerator. The film crew can select the form factor of the computer according to the needs and limitations of the film set. It can be a desktop computer, notebook, or industrial computer mounted in the rack together with other equipment. The selected computer needs to be extended with an image acquisition card(s) supporting SDI, which is a common standard used in the modern film industry. The system supports SDI input cards manufactured by Blackmagic Design company. This company offers a wide range of SDI-based solutions. Their extension cards provide from 1–8 SDI inputs and can communicate with the CPU using an Universal Serial Bus (USB) (solution preferred for notebooks) or Peripheral Component Interconnect Express (PCIe) interface (solution for desktop and industrial computers). The system can handle more than one card. Its final configuration depends on the user.

The SDI extension cards are connected to two cameras mounted in the stereoscopic rig. In general, professional cameras dedicated to cinematography store all data on removable disks attached directly to the camera. However, they are equipped with an additional SDI output for image preview. The preview image is distributed throughout the entire film set and available for all the devices such as monitors, recorders, etc. Therefore, the natural choice was to use these signals from both cameras as the inputs of the diagnostic system. These signals are easily available on the set, and their quality is enough for performed analyses.

As the professional cinematography stereoscopic system comprises two independent cameras, an important issue is their precise synchronisation, correct acquisition, and the correlation of both video streams. As shown in Figure 1, both cameras are connected to a dedicated synchronisation network distributing a signal compliant with the one of the Society of Motion Picture and Television Engineers (SMPTE) timecode standards. The SMPTE timecode standards include among others: Linear Timecode (LTC), Vertical Interval Timecode (VITC), burnt-in timecode, MIDI timecode, etc. [11,12]. The timecode network on the film set is always synchronised to a single device called a master generator and distributes its time to all cameras and audio recorders. Thanks to the information delivered to the cameras using the timecode network, all frames in the video stream are marked with the unique identification code, which contains the binary encoded hour, minute, second, and frame number. The developed system uses the information for synchronizing the incoming video streams. This process is crucial in data pre-processing, as only precisely synchronised image frames can be considered stereoscopic pairs and used for disparity map estimation.

As already mentioned, the computer used in the system should be equipped with a GPU card. Most of the modern GPU cards support general-purpose computing and can be used as a co-processor for the CPU. The General-Purpose Graphics Processing Unit (GPGPU) cards are very efficient in image processing. Therefore, some parts of the implemented algorithms use the GPU to obtain higher performance (see Section 3).

The presented architecture is relatively simple, and according to the requirements, the system is easy to build and maintain. For proper operation, it requires only two connections to camera preview outputs. As mentioned, these signals are distributed anyway throughout the film set (e.g., for film crew monitors). This approach makes the system non-invasive, significantly facilitates its use, and allows its installation far away from the rig.

## 3. Disparity Map Estimation Algorithms

**Figure 2:**
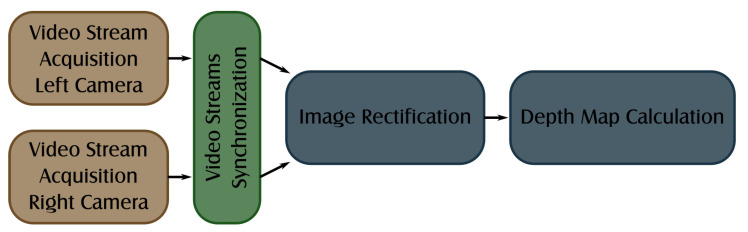
Disparity map calculation algorithm.

Determining a disparity map on a film set during the recording process allows the creators to analyse, evaluate, and if necessary, correct the quality of the 3D image. The generated disparity map should reflect the image observed by the camera as closely as possible, without distortions and clipping the image area. Therefore, the main objective of the work was to develop appropriate, efficient algorithms for image preparation (rectification) and determining a disparity map. Figure 2 presents the data flow diagram for the implemented diagnostic system. The raw data from the cameras are acquired by the two independent threads, synchronised using timecode metadata attached to each frame, and transferred to the data-processing thread that performs image rectification and disparity map calculation.

### 3.1. Rectification

The methods of stereoscopic image rectification can be divided into two main groups: calibrated and uncalibrated. The calibrated rectification methods, in addition to the input images, also require knowledge of the intrinsic (e.g., focal length, principal point) and extrinsic (e.g., mutual position and orientation) camera parameters [13,14]. Thanks to this, the methods achieve high-accuracy results for almost any stereoscopic camera. However, it should be remembered that the cameras used on a film set are very diverse, and their settings change very often. Determining the parameters mentioned above for each camera would be a long and laborious process. Therefore, for the needs of the presented diagnostic system, it was decided to use the uncalibrated rectification method.

The uncalibrated rectification methods require only two input images, which is their main advantage from this system point of view. The use of these methods means that the system can only be connected to SDI signals from cameras and does not require any additional actions from the user (such as complicated calibration procedures). The uncalibrated methods are usually based on detecting and matching the image features. This means that they are flexible, but at the same time, more complicated, and hence computationally complex and less efficient.

The review and evaluation of the available rectification algorithms showed that many are not suitable for application in a real-time diagnostic system. The authors evaluated rectification algorithms implemented in two popular image-processing frameworks—the Open Source Computer Vision (OpenCV) library and the Image Processing Toolbox for MATLAB. Both implementations are based on algorithms using a fundamental matrix to describe the epipolar geometry (see [15,16]). Another method implemented and evaluated during the research studies was based on the algorithm presented in [17]. It is also dedicated to stereoscopic convergent cameras and assumes that the epipolar lines can be used instead of the fundamental matrix for low convergence angles. The last solution considered by the authors was the implementation of the algorithm described in [18] and given in [19]. The method approximates calibrated rectification based on the minimisation of a cost function.

The research on uncalibrated rectification algorithms was conducted using 25 various example movies registered using a professional stereoscopic rig. All the above algorithms were discarded mainly because of distortions in the output image. The rectified images were often significantly distorted (rotated, scaled, and/or cropped). Another reason was the unsatisfactory processing efficiency. The execution time for full HD test images ranged from a few to several seconds per frame.

The literature study and evaluation of the selected algorithms carried out using recordings from a professional stereoscopic rig showed that the most satisfactory results were obtained using the keystone correction algorithm proposed in [20]. The algorithm is dedicated to stereoscopic systems, assuming low convergence angles. The evaluation results using the example movies showed that the algorithm produces non-distorted output with satisfactory performance. The initial implementation of the algorithm showed that the average time required to rectify the full HD frame was 2–3 s. The implemented algorithm can be divided into the following steps:

1. Features’ detection: Key points of the individual images are detected using the *Speeded-Up Robust Features (SURF)* algorithm [21] and correlated between images to create a list of key point pairs whose descriptors best match each other;
2. Removing outliers: Pairs of key points that have been assigned incorrectly in the previous step are removed from the list in order not to affect the calculations in the next steps. The deletion is based on the distance of the points in the pair and their X and Y coordinates;
3. Target features’ calculation: Target key points are calculated in such a way that the horizontal coordinate is the same as for input image, while the vertical coordinate is the average value of the original key points;
4. Features-to-mesh mapping: The image is divided into an *M × N* uniform grid mesh, and every original key point is then mapped to the appropriate mesh cell according to its coordinates;
5. Bilinear inversion: The key points (both source and target) are represented as a linear combination of the four vertices of the mesh cell;
6. Target mesh calculation: The target mesh cell calculation is defined as a linear least-squares problem that moves the key points to the target positions and minimises visual distortion. The implementation uses sparse linear system solvers available in the Eigen library [22];
7. Warping: This is the mapping pixels of the original input image to the rectified output image. The warping is guided by the mesh vertices’ position. To simplify the transformations, the forward warping method was selected for the implementation. The pixels of the output image that were not set during the mapping are calculated based on values of neighbouring pixels [23].

**Figure 3:**
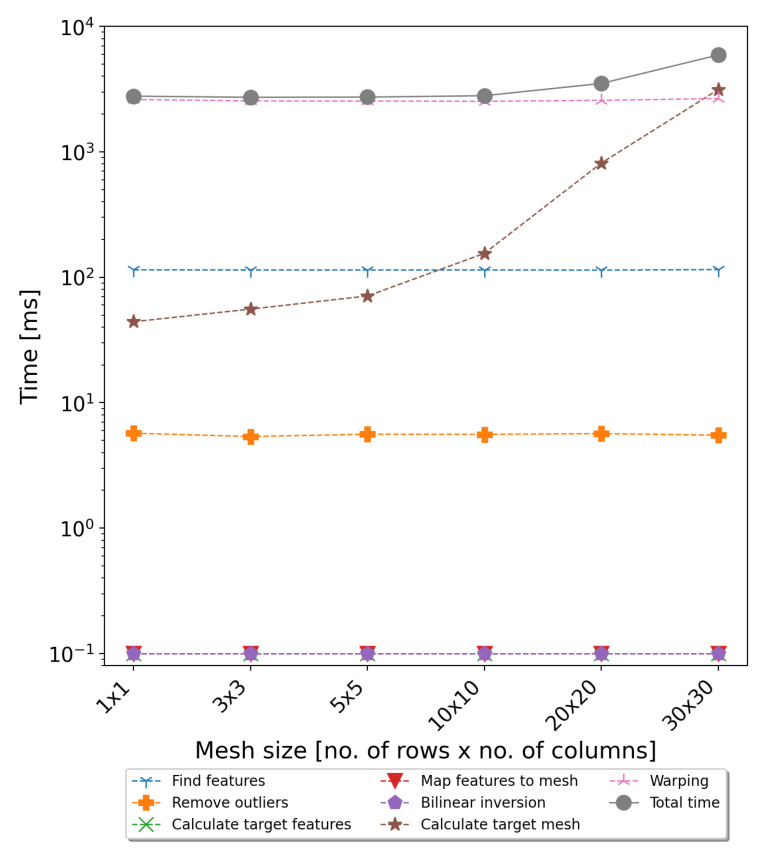
Average execution time for the rectification algorithm steps (CPU version) for various mesh sizes.

The evaluation of the execution time of the above steps showed that the most time-consuming parts of the algorithm are warping and (for dense mesh) calculating the target mesh coordinates. Detailed measurements of the execution time of all individual steps are presented in Figure 3. Taking into account that the warping is a pixelwise algorithm, it is ideal for GPGPU parallelisation.

The GPGPU version of the warping algorithm was implemented using Open Computing Language (OpenCL) to ensure portability to other operating systems and platforms. The main profit of the GPGPU implementation is to parallelise the processing of pixels into many independent threads. The size of the working group is parametrised so the application can be adapted to the hardware platform used. The algorithm code is divided into two kernels. The first is responsible for transforming pixels from an input image to an output image. It takes the input image pixels and calculates the new coordinates using target mesh vertices. Then, it copies the pixel from the input to output image and marks the output pixel coordinates in an additional mask image. The second kernel checks in the mask image which pixels in the output are not filled, and based on this information, it calculates their values using the data from neighbouring pixels. The GPU implementation was tested using two GPU cards: NVIDIA GeForce GT 650 M (384 CUDA Cores, 2048 MB GDDR5) (https://www.techpowerup.com/gpu-specs/geforce-gt-650m.c547 (accessed on: 12 December 2021)) and NVIDIA GeForce GTX 780 Ti (2880 CUDA Cores, 3072 MB GDDR5) (https://www.techpowerup.com/gpu-specs/geforce-gtx-780-ti.c2512 (accessed on 12 December 2021)). The parallelised implementation allowed achieving five-times acceleration for the first GPU card and twelve-times for the second GPU card. Detailed results are presented in Section 5.

### 3.2. Disparity Map Calculation

The final stage of image processing is the computation of the disparity map. The algorithms available in the literature were reviewed regarding the quality of the generated results and computational efficiency. The following algorithms were selected for the detailed evaluation: StereoBM [24], StereoSGBM—the modified version of StereoSGM [25] implemented in the OpenCV library—and segment-based stereo matching using belief propagation [26]. As a starting point for a CPU-only implementation, the SGBM algorithm was selected as it generates a dense disparity map with good accuracy. At the same time, the computation time is relatively short. For the test data used within the framework of the conducted works (see Section 5), it was approximately 1–2 s per frame. A processing time and latency of about 1 s could be acceptable for cursory image analysis on a movie set, but a better performance is strongly recommended.

As in the case of rectification, the acceleration of the calculations using the GPU unit was applied. The GPGPU implementation was based on the algorithm proposed in [27]. The algorithm was integrated with other components of the software. It can be executed in the GPU immediately after the rectification. This allows avoiding data copying between then CPU and GPU. Ultimately, thanks to the use of the GPU, the time needed to determine the depth map was less than 100 ms, which gives at least ten-times acceleration.

**Figure 4:**
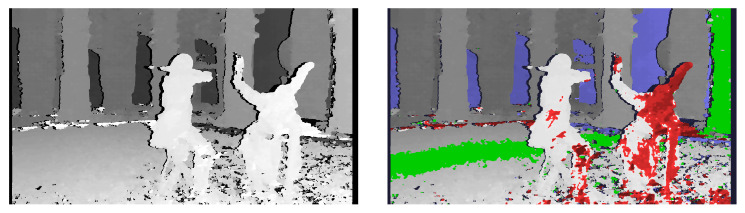
Calculated disparity map without image rectification—greyscale (**left**) and with depth budget constraints’ information (**right**).

**Figure 5:**
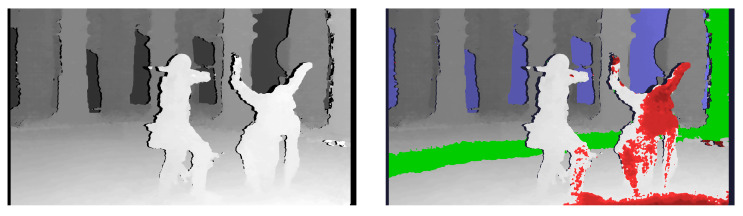
Calculated disparity map after image rectification—greyscale (**left**) and with depth budget constraints’ information (**right**).

Thanks to the acceleration of the calculations using the GPU, it is possible to process a few image frames per second, assuming that the video preview distributed over the movie set is full HD resolution. A higher frame rate makes the disparity map video feel smoother and improves the quality of work with the system. Figure 4 and Figure 5 present example outputs of the disparity map generated by the described diagnostic system. Figure 4 presents the disparity map calculated for raw images acquired from the stereoscopic rig (without rectification). Figure 5 presents the disparity map for the same images, but after rectification performed with the algorithm described in Section 3.1. The comparison of both figures shows that even for stereoscopic rigs with low-angle convergence, the rectification of the input images significantly affects the quality of the generated disparity map. It is related to the optimisation of the depth map determination algorithms, which, to save computing time, assume practically zero vertical disparity. Detailed results from the evaluation of the system performance are presented in Section 5.

## 4. Software

The algorithms described in the previous section were integrated with a uniform functional graphical application. The application is an easy-to-use tool with a Graphical User Interface (GUI) that controls all the hardware components, ensures the acquisition and synchronisation of video streams, performs data processing, and visualises the raw data and analyses the results. The software application is the only system component with which the user interacts. This means the GUI should be ergonomic and intuitive to ensure easy operation in a very dynamic environment such as a movie set. On the other hand, the software also implements all the image-processing algorithms, which need to be very efficient and robust.

### Structure of Image Acquisition and Processing Application

All the components that make up the user application can be divided into three main layers:

- Device drivers—responsible for communication with the hardware components. Device drivers provided by hardware manufacturers, together with related low-level libraries, support discovering all connected devices, communication and control, as well as the acquisition of raw data from the cameras;
- Image-processing libraries—provide all the algorithms required for camera calibration and disparity map estimation;
- GUI application—merges all lower-level components and ensures video acquisition, synchronisation, processing, and visualisation.

The application was developed for the Windows operating system and tested using Windows 7 and Windows 10. Still, one of the assumptions for future development is migration with macOS, which is commonly used in the cinema industry. Therefore, all the tools and libraries used in the developed software are supported by both operating systems. The primary development environment was the Qt toolkit. It is an open-source environment supporting the development of cross-platform applications and creating modern graphical user interfaces. The software also uses the OpenCV library for image-acquisition and -processing purposes. The library is an extensive collection of computer vision algorithms optimised for efficiency and real-time processing. Some image-processing algorithms implemented for the needs of the presented diagnostic system can be executed using a GPGPU. These algorithms were implemented using the OpenCL framework. This was selected because it ensures cross-platform compatibility. Thanks to OpenCL’s usage, the software can be executed across heterogeneous platforms, taking into account various operating systems and GPU types and architectures.

**Figure 6:**
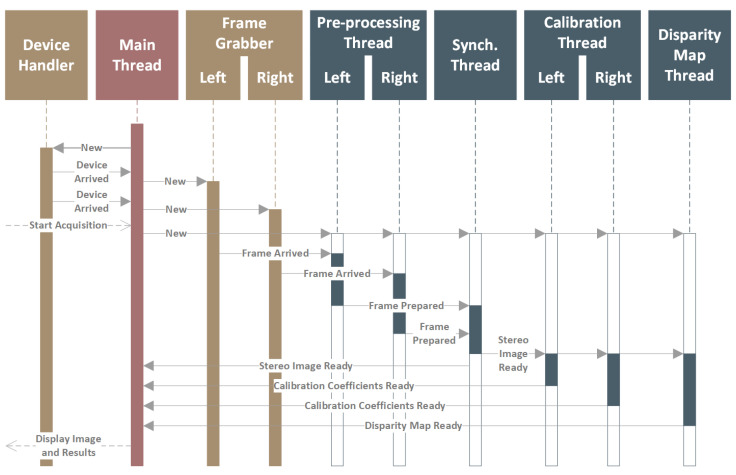
Multi-threaded execution sequence in the graphical user application for image acquisition, synchronisation, and processing.

Figure 6 shows a simplified sequence diagram presenting all the algorithms executed by the software to acquire and synchronise two video streams, process images, and display all information to the user. As shown in the diagram, the software uses multiple independent threads for individual tasks to improve performance and achieve the maximum use of the processing power of the CPU. Communication between all the threads is based on signals and callback functions, reducing the latency and facilitating software development.

The *Main Thread* of the application is responsible for handling and monitoring all events happening in the system. First, it communicates with the libraries responsible for detecting and managing the SDI input modules. Based on the information provided by these libraries, two *Frame Grabber* objects are created, one per SDI input. The objects are wrappers providing the interface for the Qt and OpenCV libraries to configure the devices and acquire a full video stream.

The acquired raw data streams from both cameras are transferred to *Pre-processing Threads*. The pre-processing includes, but is not limited to, image rotations and flipping, which are especially useful in the case of a mirror rig. After the pre-processing, both streams are transferred to the *Synchronisation Thread*. It creates stereo pair images based on the timecode information attached to each video frame. After synchronisation, both frames are considered inseparable stereo pair image, and as such, they are processed by all other application components.

The synchronised video stream can be used by the *Calibration Threads* and *Disparity Map Thread*. The operation mode of the application can be changed during the operation by selecting the appropriate view. Threads related to a given operation mode are properly started and stopped so as not to overload the processor with unnecessary tasks. The *Calibration Threads* execute the algorithms aimed at the detection of the calibration reference board (separately on the left and right image) and the calculation of the camera alignment coefficients (using results from both images) [10]. The *Disparity Map Thread* is responsible for disparity map estimation. For this purpose, it first rectifies the images using the uncalibrated rectification algorithm and then calculates the disparity map (see Section 3).

**Figure 7:**
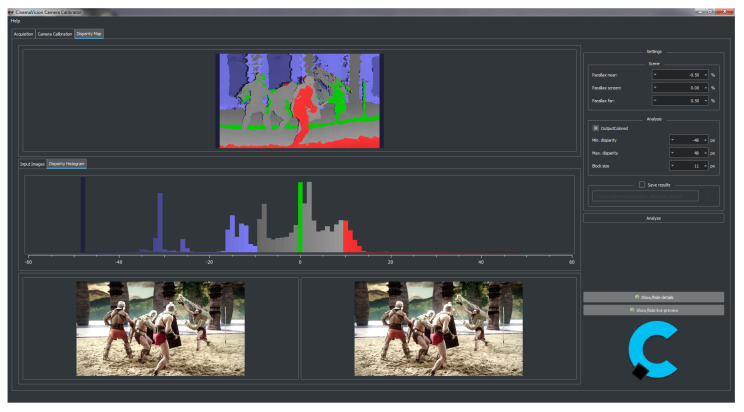
Graphical user interface for the disparity map calculation algorithm.

An exemplary view of the main application window is shown in Figure 7. On the right side, there are controls necessary for the application configuration (e.g., data acquisition devices, pre-processing configuration) and disparity map algorithm settings. The main view is divided into two sections. The top part presents the main result of the disparity map algorithm—a greyscale disparity map coloured based on the depth budget information. The middle part is the disparity histogram calculated for the disparity map. In the bottom part, the application displays a preview of raw data received from the cameras. Each of the three sections can be hidden or displayed depending on the user’s needs.

## 5. Experimental Evaluation and Results

The algorithms for the rectification and determination of the disparity map implemented in the developed system were tested in terms of functionality and performance. The functional verification was carried out mainly based on the Middlebury Stereo Datasets [28]. The aim of the tests with the use of the Middlebury Datasets was to verify the quality of the generated disparity map and, above all, to verify the rectification algorithm and its impact on the map accuracy. For this purpose, many tests were carried out using 10 evaluation training and 13 additional datasets. These datasets contain a Ground Truth (GT) image to verify the obtained results. The tests consisted of calculating the depth map for the raw image and the image after rectification using the developed algorithm. Then, the number of incorrect pixels (as a percentage) and the average error of the obtained disparity map (in pixels) were compared. To select the best mesh size that would be the best compromise between accuracy and performance, the tests were performed for different mesh dimensions—1 × 1, 3 × 3, 5 × 5, 10 × 10, 20 × 20, and 30 × 30 (rows × columns). Table 1 presents the results for the selected measurements. The underlined values indicate that the result after rectification was better than for the original image. For example, for dataset *Storage*, the number of invalid pixels in the disparity map calculated without rectification was 47.54%, and the average error in the calculated disparity was 4.48 px. Executing the rectification algorithm before calculating the disparity map reduced both indicators regardless of the mesh size. For example, for a mesh with five rows and five columns, the number of invalid (undetermined) pixels was reduced to 43.68 % and the average disparity error to 4.31 px. The results obtained for all the datasets showed that the implemented algorithm works correctly and, for meshes other than 1 × 1, positively influences the accuracy of the generated disparity map for almost all evaluated datasets. It should also be noted that increasing the mesh size improved the quality of the map.

The rectification algorithm was also tested on sample movies recorded on a real film set. It was a set of 25 films shot under various conditions, both indoors and outdoors. The videos were recorded in full HD resolution (1920 × 1080 pixels). Each movie contained approximately 150 frames. A common problem in uncalibrated rectification methods based on detecting image features is the incorrect identification of key points. This results in a wrong rectification and hence the deterioration of the correlation between the left and right image. The tests aimed to check the quality of the rectification on the pictures from the film set, and the measure was the average vertical disparity calculated for a set of selected key points before and after the rectification. The evaluation consisted of determining several dozen key points using the SURF algorithm in the right and left images of the stereoscopic pair. The points were then paired, manually verified, and outliers discarded. For the rest of the points, the average vertical disparity was calculated. The underlined values show the cases when the average vertical disparity after rectification was smaller than before. The results showed that the rectification algorithm worked correctly for almost all test videos. Only in the case of one film, the mean vertical disparity was greater after rectification. It is important that even in the case of movies shot at a low convergence angle (small disparity in the raw file), the rectification algorithm did not cause erroneous image distortions and magnification of disparity.

**Figure 8:**
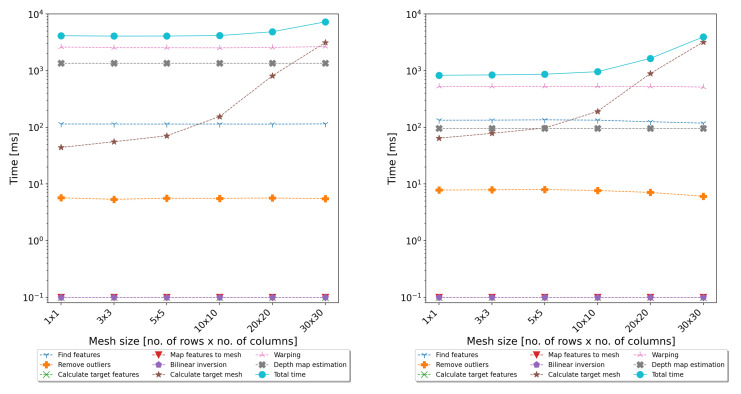
Results of the performance evaluation using the notebook computer—execution time of individual steps of the algorithms on a CPU (**left**) and GPU (**right**).

**Figure 9:**
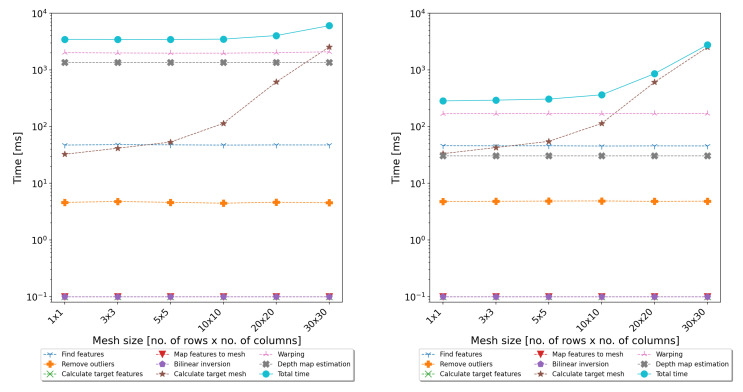
Results of the performance evaluation using the desktop computer—execution time of individual steps of the algorithms on a CPU (**left**) and GPU (**right**).

In addition to functional tests, performance tests of the full-image-processing path, including the rectification and calculation of the disparity map, were also carried out. The performance tests consisted of processing all 25 test films and measuring the execution time of individual steps of the algorithms. Two implementations of the algorithms were tested—one based only on the CPU and one also using the GPU. The tests were performed on two typical personal computers—desktop and notebook. The desktop computer was equipped with an Intel Core i7-4790 CPU, 32 GB of Random-Access Memory (RAM), and an NVIDIA GeForce GTX 780 Ti GPU card. The notebook was equipped with an Intel Core i7-3630QM CPU, 8 GB of RAM, and an NVIDIA GeForce GT 650 M GPU card. The results are presented in Figure 8 and Figure 9.

**Figure 10:**
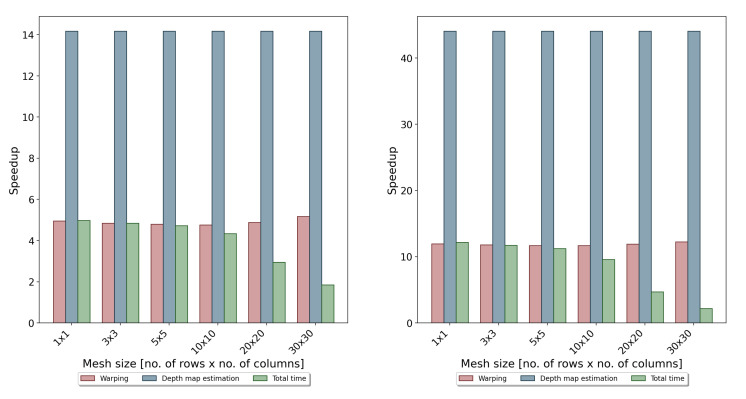
Results of the performance evaluation—speedup obtained by using the parallelisation of the algorithms using a GPU: results for a notebook (**left**) and desktop (**right**).

As can be seen, the use of the GPU allowed significantly reducing the time of warping and determining the disparity map. The resulting acceleration is shown in Figure 10.

In the case of the notebook, the obtained acceleration was 4–5-times, and the average processing time required to calculate the disparity map was about 1 s per frame. In the case of the desktop computer with much better performance parameters, the speedup was about 10–12-times, and the total processing time was around 300 ms. As shown in Figure 8 and Figure 9, the last non-optimised part was the algorithm responsible for calculating the coordinates of the target mesh vertices. Its execution time is related to the mesh size, and for dense meshes, it can significantly slow down the whole algorithm. However, as can be seen from Table 1 and Table 2, the use of fine meshes did not bring significant advantages in terms of accuracy. For the tested mesh sizes, the 10 × 10 size seemed to compromise the accuracy and efficiency of the proposed algorithms. However, in the further development of the system, the authors considered also transferring this step to the GPU and using a dedicated sparse matrix solver.

The proposed diagnostic system was evaluated on film sets during the production of 3D films (in cooperation with the Polish companies FINN Sp. z o.o. and Isyrius). It was tested with two different camera models: EPIC MYSTERIUM-X manufactured by RED Digital Cinema and Phantom Flex4K from Vision Research. The system correctly acquired and synchronised the video streams for both of them. The developed method implemented in the prototype system gave results satisfactory for the film crew and allowed the detection of possible problems in the recorded image. It is possible to assess whether the recorded stereoscopic material is correct, does not violate the depth budget, and meets the assumptions for a given scene. An exemplary window with system processing results is shown in Figure 7. It can be seen that the characters in the foreground exceed the depth budget assumed for the given scene and camera configuration. Such a violation may cause difficulties with the image reception of the viewer due to the need to squint.

## 6. Conclusions

The main result of the research presented in this paper was the efficient GPU-based implementation of algorithms dedicated to the support of the film crew in analysing the quality of the recorded stereoscopic image. The proposed solution allows near real-time determining of the 3D disparity map in parallel with the image-recording process. The method was implemented and evaluated in the prototype diagnostic system dedicated to being used directly on the film set to acquire, synchronise, and process the video streams from stereoscopic converged rigs.

The implemented algorithms were tested as part of the automated system developed for monitoring the 3D image quality on a film set. The main goal of the system is to provide a disparity map and histogram with the best possible reflection of the recorded image. The proposed approach and high-performance implementation of the algorithms using a GPU support allowed the development of the diagnostic system, which can generate and mark a disparity map on an ongoing basis. Its advantage is the lack of a need to calibrate and know the parameters of the cameras, thanks to which it easily adapts to frequent changes on the film set. The implementation of the selected algorithms using a GPGPU accelerator resulted in a 10-fold acceleration of the calculations and a reduction in the time for determining the disparity map to 300 ms. The processing efficiency at the level of a few frames per second (at full HD resolution) allows for relatively comfortable work with the system and tracking the image quality in parallel with its recording. Thanks to the uncalibrated image rectification algorithm, the system easily adapts to frequent changes of the camera configuration, which makes it easy to use and non-invasive in the entire filming process.

The developed universal graphic software can be used on any hardware platform, enabling it to be used in various environments and film sets. However, the performance evaluation showed that it is still possible to optimise the processing algorithms, especially those related to image transformation and solving sparse matrix equation systems. It is also worth considering migrating and testing the developed algorithms on modern miniaturised GPUs (such as NVIDIA Xavier). The embedded GPU modules can further miniaturise the entire system and extend its scope of application.

## Figures and Tables

**Table 1:** Results of the accuracy evaluation of the proposed algorithms using the Middlebury Stereo Datasets [28].

	Raw		Rectified Using Mesh Size							
			1 × 1		5 × 5		10 × 10		30 × 30	
Dataset	Invalid [%]	Avg. Error [px]	Invalid (%)	Avg. Error (px)	Invalid (%)	Avg. Error (px)	Invalid (%)	Avg. Error (px)	Invalid [%]	Avg. Error [px]
Adirondack	37.75	6.77	38.63	7.49	36.29	6.91	36.24	6.89	36.38	6.96
Backpack	8.99	1.68	9.05	1.72	8.64	1.65	8.65	1.67	8.82	1.71
Bicycle1	42.92	11.9	40.69	11.78	42.07	13.65	39.38	13.19	41.6	11.61
Cable	24.78	3.39	28.21	3.41	21.47	2.94	22.04	2.91	23.24	3.32
Classroom1	17.57	1.04	15.56	1.06	15.75	1.02	15.97	1.04	16.3	0.99
Couch	27.59	3.96	27.58	4.29	27.48	4.25	27.48	4.28	27.47	4.25
Flowers	47.42	3.41	48.75	3.88	45.09	3.63	44.7	3.19	45.39	3.29
Jadeplant	35.43	4.9	35.0	5.14	35.87	5.13	35.67	5.2	34.98	5.1
Mask	26.5	3.07	26.6	3.36	26.53	3.41	26.43	3.38	26.29	3.38
Motorcycle	14.31	1.62	11.13	1.29	11.41	1.35	11.81	1.4	12.78	1.54
Piano	20.83	3.61	21.12	4.21	20.15	4.21	20.07	4.17	20.1	4.17
Pipes	14.92	2.38	10.39	2.2	11.13	2.26	11.44	2.29	13.28	2.42
Playroom	34.74	5.8	33.64	5.96	34.24	6.04	33.91	5.99	33.96	5.95
Playtable	30.05	7.91	18.76	1.97	19.08	2.01	25.47	5.29	29.27	7.68
Recycle	22.78	1.71	22.5	1.82	22.13	1.79	21.73	1.73	22.08	1.78
Shelves	43.16	3.97	41.4	3.3	40.52	3.58	41.65	3.79	41.61	3.9
Shopvac	64.84	12.13	64.92	13.3	66.25	13.32	64.93	12.94	64.87	13.48
Sticks	11.83	2.05	11.83	2.2	11.7	2.18	11.68	2.19	11.68	2.2
Storage	47.54	4.48	42.88	3.95	43.68	4.31	43.83	4.38	45.31	4.39
Sword1	10.65	1.33	10.64	1.38	10.44	1.36	10.38	1.38	10.32	1.38
Sword2	46.12	9.16	43.27	5.92	34.91	5.1	37.34	6.34	41.66	8.13
Umbrella	39.42	4.08	37.61	4.12	37.63	4.03	37.87	4.07	38.51	4.25
Vintage	46.29	3.88	45.72	3.98	46.13	4.1	45.82	4.0	45.77	4.04

**Table 2:** Average value of vertical disparity (in px) for all frames in the movie—before (raw) and after (rectified) rectification.

Movie	Raw	Rectified					
		1 × 1	3 × 3	5 × 5	10 × 10	20 × 20	30 × 30
01	2.6	2.6	2.5	2.6	2.6	2.5	2.5
02	0.7	0.9	0.7	0.6	0.6	0.6	0.6
03	11.9	1.1	0.8	0.9	1.3	2.0	2.6
04	0.7	0.6	0.6	0.6	0.6	0.5	0.5
05	6.4	0.9	0.8	0.8	0.9	1.3	1.7
06	28.8	2.8	2.8	3.0	3.9	6.3	9.8
07	5.1	1.2	1.0	1.1	1.3	1.7	1.9
08	0.4	0.6	0.4	0.4	0.4	0.4	0.4
09	0.7	1.0	0.4	0.4	0.4	0.4	0.4
10	2.9	0.8	0.8	0.8	0.7	0.7	0.8
11	1.0	0.5	0.4	0.4	0.4	0.4	0.4
12	0.6	1.0	0.8	0.8	0.7	0.7	0.7
13	4.2	0.7	0.5	0.3	0.3	0.4	0.4
14	0.9	0.8	0.6	0.5	0.4	0.4	0.4
15	0.6	0.7	0.5	0.5	0.4	0.5	0.5
16	2.4	0.9	0.6	0.5	0.5	0.5	0.6
17	1.3	0.7	0.7	0.7	0.7	0.8	0.8
18	1.8	0.7	0.5	0.6	0.5	0.5	0.6
19	7.5	1.2	1.1	1.1	1.2	1.9	2.8
20	0.5	1.0	0.5	0.4	0.4	0.4	0.4
21	2.7	2.3	2.4	1.9	1.6	1.6	1.7
22	0.8	0.6	0.4	0.4	0.3	0.4	0.4
23	85.5	0.4	0.4	0.4	0.5	2.3	7.4
24	0.5	0.5	0.5	0.5	0.5	0.5	0.5
25	0.3	0.5	0.3	0.3	0.3	0.3	0.3

## Abbreviations

The following abbreviations are used in this manuscript:

2D	Two-Dimensional
3D	Three-Dimensional
SDI	Serial Digital Interface
USB	Universal Serial Bus
CPU	Central Processing Unit
FPGA	Field-programmable Gate Array
GPU	Graphics Processing Unit
GPGPU	General-Purpose Graphics Processing Unit
PCIe	Peripheral Component Interconnect Express
API	Application Programming Interface
SDK	Software Development Kit
SIMD	Single Instruction, Multiple Data
OpenCL	Open Computing Language
GUI	Graphical User Interface
IDE	Integrated Development Environment
SMPTE	Society of Motion Picture and Television Engineers
RAM	Random-Access Memory
